# Linking Non-alcoholic Fatty Liver Disease Severity With Metabolic Syndrome Features: An Integrative Study on Clinical and Radiological Fronts

**DOI:** 10.7759/cureus.59788

**Published:** 2024-05-07

**Authors:** M Sukumar, Naval K Vikram, Piyush Ranjan, M Pandey, Ashu S Bhalla, Lakshmy Ramakrishnan, Danish Javed, Varun Malhotra, Roshan Prasad, Gaurav Mittal

**Affiliations:** 1 General Medicine, All India Institute of Medical Sciences, Bhopal, IND; 2 Medicine, All India Institute of Medical Sciences, New Delhi, IND; 3 Biostatistics, All India Institute of Medical Sciences, New Delhi, IND; 4 Radiodiagnosis and Interventional Radiology, All India Institute of Medical Sciences, New Delhi, IND; 5 Cardiac Biochemistry, All India Institute of Medical Sciences, New Delhi, IND; 6 Ayurveda, Yoga, Naturopathy, Unani, Siddha, and Homeopathy (AYUSH), All India Institute of Medical Sciences, Bhopal, IND; 7 Physiology, All India Institute of Medical Sciences, Bhopal, IND; 8 Medicine and Surgery, Jawaharlal Nehru Medical College, Datta Meghe Institute of Higher Education and Research, Wardha, IND; 9 Internal Medicine, Mahatma Gandhi Institute of Medical Sciences, Wardha, IND; 10 Research and Development, Student Network Organization, Mumbai, IND

**Keywords:** asian indian population, subclinical inflammation, insulin resistance, bone mineral density, nafld

## Abstract

Introduction

Non-alcoholic fatty liver disease (NAFLD) has become a widespread cause of chronic liver disease, ranging from simple steatosis to severe conditions like non-alcoholic steatohepatitis (NASH) and cirrhosis. Despite its similarity to alcohol-induced liver damage, NAFLD affects individuals with no significant alcohol consumption. This study explores the association between NAFLD, bone mineral density (BMD), insulin resistance, and subclinical inflammation, focusing on the Asian Indian population. The primary objective was to investigate the relationship between NAFLD and BMD, insulin levels, and markers of subclinical inflammation, hypothesizing that patients with NAFLD exhibit lower BMD, possibly linked to insulin resistance and inflammation.

Methodology

A cross-sectional study with 100 subjects aged 18-50 years (50 cases with NAFLD and 50 controls) was conducted. Exclusion criteria included excessive alcohol consumption, drug-induced fatty liver, severe organ dysfunction, infections, pregnancy, and acute or chronic illness. Data were collected through clinical examinations, anthropometric measurements, biochemical investigations, ultrasound diagnosis of NAFLD, and dual-energy X-ray absorptiometry (DEXA) scans for BMD assessment. Statistical analysis employed the chi-squared tests, t-tests, and Wilcoxon rank-sum tests.

Results

NAFLD patients had higher body mass index (BMI), waist-to-hip ratio, and markers of insulin resistance and inflammation compared to non-NAFLD controls. DEXA scans revealed significantly lower BMD in NAFLD cases, along with a higher prevalence of osteopenia. Positive correlations were observed between BMD and insulin resistance. The study contributes to understanding the link between NAFLD and lower BMD in the Asian Indian population, emphasizing the impact of insulin resistance and inflammation on bone health. The literature review supports the relevance of exploring NAFLD as an independent risk factor for low BMD.

Conclusion

This case-control study underscores the significant association between NAFLD and lower BMD in the Asian Indian population. Despite limitations, the findings highlight the importance of further research with larger samples and comprehensive assessments to elucidate the interplay between NAFLD, metabolic factors, and bone health.

## Introduction

Non-alcoholic fatty liver disease (NAFLD) has emerged as a prevalent cause of chronic liver disease, ranging from simple fatty infiltration (steatosis) to more severe conditions like non-alcoholic steatohepatitis (NASH) and cirrhosis [1]. Despite its pathology resembling alcohol-induced liver damage, NAFLD occurs in individuals who do not consume harmful amounts of alcohol. Diagnosing NAFLD involves excluding excessive alcohol intake and other causes of fatty liver, often discovered incidentally during investigations of elevated liver enzyme levels [1]. NAFLD is now recognized as part of the metabolic syndrome, strongly associated with adiposity and insulin resistance [2]. The prevalence of NAFLD is alarming, affecting 20-30% of the population in Western countries, reaching 70-90% among those with obesity or diabetes [3]. In countries like India, the rising prevalence of diabetes, obesity, and insulin resistance contributes significantly to the increasing incidence of NAFLD [4]. Diabetes mellitus, obesity, and hyperinsulinemia are commonly linked with NAFLD [5]. The hepatic manifestation of metabolic syndrome, NAFLD, closely correlates with hepatic insulin resistance and liver enlargement [5]. Various radiological investigations, with liver biopsy being the gold standard, can evaluate the extent of liver involvement in metabolic syndrome [1]. Patients with NAFLD often exhibit insulin resistance, low-grade inflammation, and altered markers of bone metabolism [6,7]. There is evidence suggesting an increased risk of osteoporotic fractures in NAFLD, possibly due to chronic inflammation, vitamin D deficiency, and limited physical activity [8].

We hypothesize that patients with NAFLD have lower bone mineral density (BMD) than those without NAFLD, potentially related to insulin resistance and subclinical inflammation. This study aims to assess BMD, insulin levels, and markers of subclinical inflammation, comparing these parameters between patients with and without NAFLD. To support these assertions, previous studies have emphasized the global epidemiology of NAFLD and its association with type 2 diabetes [4], the proposed nomenclature for metabolic-associated fatty liver disease (NAFLD) [2], and the diagnosis and management guidance from the American Association for the Study of Liver Diseases [1].

Furthermore, research has highlighted the clinical implications of NAFLD as a novel cardiometabolic risk factor for type 2 diabetes [6] and its association with contributors to secondary osteoporosis and metabolic bone diseases [7,8]. Studies also explored the negative association between fat mass and bone mineral content [9,10], adding valuable insights to our hypothesis. In the subsequent sections, we will delve into a comprehensive examination of BMD, insulin levels, and markers of subclinical inflammation, aiming to provide a clearer understanding of the interplay between NAFLD and these parameters.

## Materials and methods

Study design and setting

A case-control design was employed to investigate the relationship between NAFLD and BMD in adults. The study was conducted at the Outpatient Department (OPD) of AIIMS Medicine. Recruitment, exposure assessment, follow-up, and data collection occur from 2013 to 2014. Consent was obtained or waived by all participants in this study. The Institute Ethics Committee of All India Institute of Medical Sciences, New Delhi, issued approval IESC/T-73/01.02.2013.

Participants

Eligibility Criteria and Case Ascertainment

Individuals aged 18-50 years were eligible for inclusion. Cases were diagnosed with NAFLD via transabdominal ultrasound at the OPD, whereas controls were selected from individuals free of NAFLD as determined by ultrasonography. Exclusion criteria included daily alcohol consumption exceeding 20 g/day, use of liver-affecting drugs, severe vital organ dysfunction, certain infections, pregnancy, family history of liver diseases, and acute or chronic illnesses. Cases and controls were matched based on age and gender distribution. Each case was matched with a corresponding control.

Sample Size Calculation

The sample size was estimated using the OpenEpi 3.01 statistical software (Centers for Disease Control and Prevention (CDC), Atlanta, USA) with the assumptions as the confidence level of 95%, alpha of 0.05, and power of study as 80%. The minimum sample size was 136, with a 1:2 case-to-control ratio according to the statistical software. Hence, 50 cases and 100 age-matched were recruited as per a random sampling technique. 

Data sources and measurement of variables

Clinical evaluations comprised detailed history, physical examination, and anthropometric measurements. Body composition analysis was done using a Tanita body fat analyzer. Biochemical studies were conducted on venous blood samples. NAFLD diagnosis was done via transabdominal ultrasound. BMD measurement was done using dual-energy X-ray absorptiometry (DEXA) scan.

Anthropometric Measurements

Anthropometric measurements were taken according to the CDC National Health and Nutrition Examination Survey (NHANES) Anthropometry Procedures Manual. Height and weight were recorded to the nearest 0.1 cm and 0.1 kg with a stadiometer and electronic scale, respectively. Body mass index (BMI) was calculated by $BMI = Weight / (Height) ^{2}$. Waist circumference was measured midway between the iliac crest and the lowermost margins of the ribs. Hip circumference was measured at the maximum circumference of the buttocks, and the mean of three readings was taken to calculate the waist-to-hip ratio. Mid-arm circumference was measured midway between the tip of the acromion and the olecranon process. Mid-thigh circumference was measured midway between the greater trochanter and the lateral condylar process of the femur. Skin fold thickness was measured with Lange skin fold calipers to the nearest 1 mm, and the mean of three readings was recorded at each of the sites, including subscapular, suprailiac, biceps, and triceps. 

Body Fat Composition

The Tanita body fat analyzer is a novel device to estimate body fat based on the principles of bioelectrical impedance. The subjects stand barefoot on a metal sole plate that incorporates the electrodes; hence, impedance is measured through the legs and lower trunk. The underlying principle of the methodology is that the length2/impedance (H2/I) of any conducting medium is proportional to the volume of that conducting medium. Tanita TBF-305 (Tanita Corp., Tokyo, Japan) is a commercially available foot-to-foot bioelectrical impedance analysis (BIA) system. The manufacturer-supplied equations incorporate gender, mass, height, activity, category, and a measured impedance value to determine (%) body fat (100). The resistance (R) of a length of homogeneous conductive material of uniform cross-sectional area is proportional to its size (L) and inversely proportional to its cross-sectional area (A).

Biochemical Investigations

Subjects were instructed to report to the clinic after an overnight fast of at least 10 hours. Fifteen milliliters of venous blood was obtained for biochemical analysis. Complete hemograms and liver and kidney function tests were done from the routine lab. Fasting and postprandial blood sugar levels, lipid profile, high-sensitivity C-reactive protein (hs-CRP), and fasting insulin were performed on all the subjects. The blood glucose was estimated colorimetrically using a GOD-PAP test kit (Randox Lab., Crumlin, UK). The triglycerides were measured colorimetrically using a GPO-PAP test kit (Randox Lab., Crumlin, UK). Serum total cholesterol was measured colorimetrically using an enzymatic endpoint method kit (Randox Lab., Crumlin, UK). High-density lipoprotein (HDL) cholesterol was measured using a micro method to determine total cholesterol in a modified way. Hs-CRP was measured using a commercially available reagent kit based on the principle of a solid-phase enzyme-linked immunosorbent (sandwich-type) assay (solid-phase ELISA). The system utilized two highly specific monoclonal antibodies, namely, a monoclonal antibody specific for C-reactive protein (CRP) immobilized onto the microwell plate and another monoclonal antibody specific for a different region of CRP, which are conjugated to horseradish peroxidase (HRP). CRP from the sample and standards are allowed to bind to the plate, washed, and subsequently incubated with the HRP conjugate. After a second washing step, the enzyme substrate is added. The enzymatic reaction is terminated by the addition of the stopping solution. The absorbance is measured on a microtitre plate reader. The intensity of the color formed by the enzymatic reaction is directly proportional to the concentration of CRP in the sample. Fasting insulin assay was measured using a commercially available reagent kit based on the principle of enzyme-labeled chemiluminescent immunometric assay.

The solid phase (bead) is coated with monoclonal murine anti-insulin antibody. The liquid phase consists of alkaline phosphatase (bovine calf intestine) conjugated to polyclonal sheep anti-insulin antibody and alkaline phosphatase (bovine calf intestine) conjugated to monoclonal murine anti-insulin antibody. The patient sample (200 µL serum) and the reagent are incubated with the coated bead for 60 minutes. During this time, insulin in the sample from the antibody sandwich complex with the monoclonal murine anti-insulin antibody on the bead, enzyme-conjugated polyclonal sheep anti-insulin antibody, and enzyme-conjugated monoclonal murine anti-insulin antibody were present in the reagent. Unbound patient samples and enzyme conjugates are then removed by centrifugal washes. Finally, the chemiluminescent substrate containing the bead is added to the test unit, and the signal is generated in proportion to the bound enzyme.

Ultrasound of the Abdomen and Grading of Fatty Liver

Transabdominal ultrasonography was conducted by a blinded radiologist using the following standardized procedure. Transabdominal ultrasonography of the liver was done using a 3.5 MHz convex transducer (Philips Medical) using subcostal and intercostal approaches. The radiologist was blinded to the clinical data of the patients. Diffuse homogeneous increased echogenicity of the liver was diagnosed as fatty liver. The degree of fatty infiltration of the liver was graded by ultrasound. The fatty liver was graded by the following classification: (a) mild with a minimal diffuse increase in echogenicity, normal visualization of the diaphragm, and intrahepatic vessel borders, (b) moderate with a moderate diffuse increase in hepatic echogenicity with slightly impaired visualization of the diaphragm and intrahepatic vessel borders, and (c) severe with a marked increase in echogenicity, poor penetration of the posterior segment of the right lobe, and inadequate or non-visualization of blood vessel borders.

DEXA Scan

BMD was measured using DEXA, a well-established method for assessing bone density. The scan focused on specific bones, typically the spine, hip, and wrist, and compared the results against age, sex, and size-based indices. DXA, previously DEXA, measures BMD. Two X-ray beams with different energy levels are aimed at the patient's bones. When soft tissue absorption is subtracted, the BMD can be determined from the bone absorption of each beam. DEXA is the most widely used and thoroughly studied bone density measurement technology. The DXA scan is typically used to diagnose and follow osteoporosis, as contrasted to the nuclear bone scan, which is sensitive to certain metabolic diseases of bones in which bones attempt to heal from infections, fractures, or tumors. While there are many different types of BMD tests, all are non-invasive. Most tests differ in which bones are measured to determine the BMD result. DEXA is currently the most widely used, but ultrasound has been described as a more cost-effective approach to measure bone density.

Data analysis

Statistical analysis was performed using Stata Statistical Software: Release 12 (2011; StataCorp LLC, College Station, Texas, USA). Categorical data were compared using chi-squared or Fisher's exact tests, while continuous variables were analyzed using t-tests or Wilcoxon rank-sum tests, considering the distribution of the data. Statistical methods, including confounding control, subgroup analysis, missing data handling through sensitivity analysis and imputation, appropriate matching of cases and controls, and sensitivity analyses for robustness, were employed.

## Results

A total of 150 such patients were screened, of which 10 patients had diabetes, 15 had consumed alcohol, two patients were on anti-epileptic medicines, three had hepatitis B virus (HBV) infection, five patients had chronic medical illnesses, two were pregnant, and 13 refused to participate in the study. After these, patients underwent transabdominal ultrasonography for diagnosing and grading NAFLD. Of these, 50 patients had fatty liver disease who were the cases, and the rest had normal abdominal ultrasonography, who were the controls. Anthropometric parameters and blood profiles were done in 100 patients. These patients underwent clinical evaluation, anthropometric measurements, bioimpedance analysis, DEXA scan, and relevant blood investigations.

The mean age of patients with NAFLD was 36.34±8.02, similar to that of the controls, i.e., 33.88±7.24 (p=0.11). Gender distribution was also identical, with 23 males in cases and 17 males in controls (p±). Obesity and overweight were significantly higher in patients with NAFLD (84(%) versus 26(%), p<0.001).

On clinical evaluation, cases were found to have a significantly higher pulse rate systolic and diastolic blood pressure than controls, and these patients with NAFLD had similar height, waist, and hip circumference to that of controls but had higher weight, higher mid-arm and mid-thigh circumference, and higher skin fold thicknesses than controls as shown in Table 1.

**Table 1:** Baseline clinical and anthropometric parameters in NAFLD and non-NAFLD patients. NAFLD: non-alcoholic fatty liver disease; BMI: body mass index; W/H: waist-to-height ratio; cm: centimeters; mm: millimeters; mm Hg: millimeters of mercury

Parameters	NAFLD n (50), mean (SD)	Non-NAFLD n (50), mean (SD)	P-value
Height (cm)	162.02±9.29	163.88±6.25	0.24
Weight (kg)	76.23±16.60	61.768±8.96	<0.001
BMI (kg/m^2^)	28.66±4.22	22.83±2.90	<0.001
BMI 25-30	23 (46%)	12 (24%)	
BMI >30	19 (38%)	1 (2%)	<0.001
Pulse rate (bpm)	80.58±5.92	76.22±4.62	0.001
Systolic blood pressure (mm Hg)	124.28±8.19	117.7±6.49	<0.001
Diastolic blood pressure (mm Hg)	83.58±5.45	77.36±6.70	<0.001
Waist circumference (cm)	77.26±22.55	71.98±17.98	0.19
Hip circumference (cm)	79.8±23.78	78.34±20.13	0.74
W/H ratio	0.98±0.12	0.92±0.08	0.009
Mid-arm circumference (cm)	29.17±5.78	24.68±3.91	<0.001
Biceps skin fold thickness (mm)	18.47±7.82	10.32±3.88	<0.001
Mid-thigh circumference (cm)	40.41±8.95	31.8±6.51	<0.001
Triceps skin fold thickness (mm)	20.62±6.41	11.72±5.50	<0.001
Subscapular skin fold thickness (mm)	22.16±8.40	13.73±6.81	<0.001
Suprailiac skin fold thickness (mm)	23.61±7.72	16.08±5.80	<0.001

The baseline hemogram revealed significantly higher hemoglobin values in the non-NAFLD arm (13.46±1.17 vs. 12.80±1.47, p=0.01). Total leucocyte count and platelet count were similar in both arms. Bilirubin was significantly higher in the NAFLD arm as compared with non-NAFLD arm (0.64±0.32 vs. 0.48±0.16, p<0.001). Transaminitis was significantly more frequent in the NAFLD arm, as shown in Table 2. It also shows that there were significant differences in fasting blood glucose (FBG), postprandial blood glucose (PPBG), total cholesterol, HDL, low-density lipoprotein (LDL), and triglyceride parameters between patients with and without NAFLD, except for fasting insulin levels (8.9 vs. 5.7, p=0.08). There were statistically significant differences in subclinical inflammation as measured by high-sensitivity C-reactive protein (hs-CRP) (4.2 vs. 2.04, p<0.001), high erythrocyte sedimentation rate (ESR) (30(%) vs. 8(%), p= 0.009), and insulin resistance (0.26 vs. 0.16, p<0.001) as measured by the homeostasis model assessment of insulin resistance (HOMA-IR) between those with NAFLD and those without NAFLD.

**Table 2:** Hematological and biochemical characteristics of NAFLD and non-NAFLD patients. ESR: erythrocyte sedimentation rate; IU: international unit; FBG: fasting blood glucose; PPBG: postprandial blood glucose; CRP: C-reactive protein; HOMA-IR: homeostasis model assessment of insulin resistance; HDL: high-density lipoprotein; LDL: low-density lipoprotein

Parameter	NAFLD mean±SD	Non-NAFLD mean±SD	P-value
Hemoglobin (g/dL)	12.80±1.47	13.46±1.17	0.01
Total leucocyte count (cells/mm^3^)	7.6±1.6	7.38±2.04	0.52
Platelet count (c/mm^3^)	3.86±5.87	2.92±3.36	0.321
ESR (mm 1st hr)	15.6±10.10	10.64±5.87	0.01
Protein (g/dL)	7.35±0.08	7.56±0.05	0.055
Albumin (g/dL)	4.53±0.47	4.44±0.38	0.30
Bilirubin (mg/dL)	0.64± 0.32	0.48±0.16	<0.001
Serum glutamic-oxaloacetic transaminase (IU)	36.6±27.01	28.4±6.59	0.063
Serum glutamic pyruvic transaminase (IU)	28.4±6.59	28.2±9.04	0.008
Serum glutamic-oxaloacetic transaminase >50 IU/L	8/50 (16%)	0/50 (0%)	0.003
Serum glutamic pyruvic transaminase >50 IU/L	14/50 (28%)	0/50 (0%)	<0.001
Alkaline phosphatase (IU)	182.30±64.59	179.06±63.30	0.800
FBG test (mg/dL)	85.18±12.56	74.76±9.00	<0.001
PPBG test (mg/dL)	118.70±13.25	112.38±7.52	0.004
Total cholesterol (mg/dL)	181.9±6.19	150.4±4.20	0.001
HDL (mg/dL)	46.62±9.317	40.26±6.62	<0.001
LDL (mg/dL)	106.07±34.17	80.40±28.29	<0.001
Triglyceride (mg/dL) mean	168.58±69.83	138.14±25.92	<0.001
High ESR (>15 mm/hr in males, >20 mm/hr in females)	15 (30%)	4 (8%)	0.009
CRP (mg/L) median (min-max)	4.2 (0.4-19.34)	2.04 (0.25-13.64)	<0.001
High CRP >3 mg/dL	33 (66%)	20 (40%)	0.009
Fasting insulin (IU/µL) median (min-max)	8.9 (0.2-115.6)	5.7 (0.2-37.2)	0.08
HOMA-IR	0.22 (0.05-0.68)	0.17 (0.03-0.32)	0.004

The body composition was measured by the bioelectrical impedance method and tabulated in Table 3. There were no statistically significant differences between the two groups with respect to fat-free mass (FFM), total body water (TBW), and body impedance. The fat mass and fat (%) were significantly higher in patients with NAFLD as compared with those without, with a p-value of 0.001, respectively. 

**Table 3:** Body fat composition parameters in NAFLD and non-NAFLD patients. FFM: fat-free mass; TBW: total body water; NAFLD: non-alcoholic fatty liver disease

Parameter	NAFLD n, mean (SD)	Non-NAFLD n, mean (SD)	P-value
Fat %	22.62±9.88	12.89±7.37	0.001
Fat mass (kg)	22.62±9.89	12.89±7.38	<0.001
FFM (kg)	51.27±9.09	48.70±6.42	0.107
TBW	53.14±21.06	60.65±8.69	0.200

Metabolic syndrome was significantly more prevalent in patients with NAFLD compared to those with non-NAFLD (34(%) vs. 4(%), p<0.001). Impaired fasting glucose, raised triglycerides, and high blood pressure were also significantly more frequent in patients with NAFLD, as shown in Table 4. 

**Table 4:** Metabolic syndrome in these cases and controls. HDL: high-density lipoprotein

Parameters	Cases (50)	Controls (50)	P-value
Increased waist circumference >90 cm in males, >80 cm in females	20 (40%)	11 (22%)	0.05
Blood pressure ≥130/85 mm Hg	28 (56%)	6 (12%)	<0.001
Triglycerides >150	26 (52%)	10 (20%)	0.001
Low HDL	20 (40%)	30 (60%)	0.046
Impaired fasting glucose	5 (10%)	1 (2%)	0.204
Metabolic syndrome	17 (34%)	2 (4%)	<0.001

BMD measured as BMD by whole-body DEXA scan was significantly less in patients with NAFLD (1.09±0.09 vs. 1.15±0.15, p±0.02). T-score was found to be significantly lower in the NAFLD group (-0.706 vs. -0.332, p=0.0357). There were 24 patients with osteopenia in the NAFLD group (30(%) vs. 18(%)); however, the difference was non-significant. One patient of NAFLD had osteoporosis, as shown in Table 5. 

**Table 5:** DEXA scan results of NAFLD cases and non-NAFLD controls. BMD: bone mineral density; DEXA: dual-energy X-ray absorptiometry; NAFLD: non-alcoholic fatty liver disease

Parameters	Cases	Controls	P-value
BMD	1.09 (0.09)	1.15 (0.15)	0.02
T-score	-0.706 (-2.7 to 2.3)	-0.332 (-2.5 to 2.2)	0.0357
Z-score	-0.244 (-2.7 to 1.9)	-0.346 (-2.5 to 1.8)	0.5008
T-score	1/50 (2%)	0/50 (0%)	1.0
T-score -1.0 to -2.5	25/50 (50%)	14/50 (28%)	0.039

Table 6 shows the correlation of DEXA parameters with fasting insulin. Comparison between both groups shows no significant correlation in cases and controls in bone mineral content, BMD, and T-score.

**Table 6:** Correlation of DEXA parameters with fasting insulin in cases and controls. T.BMD: total bone mineral density; T.BMC: total bone mineral capacity; DEXA: dual-energy X-ray absorptiometry

Variable	Cases (n±50)			Controls (n±50)		
	Pearson's coefficient		P-value	Pearson's coefficient		P-value
T.BMC	-0.086	0.394		-0.108	0.456	
T.BMD	-0.05	0.559		-0.025	0.864	
T-score (bone)	0.157	0.119		0.16	0.27	
Z-score (bone)	0.163	0.119		0.17	0.24	

Table 7 shows the correlation of the DEXA scan parameters with HOMA-IR. There was no significant correlation of DEXA parameters with T.BMC, T.BMD, T-score, and Z-score in both cases and controls. 

**Table 7:** Correlation of DEXA parameters with HOMA-IR in cases and controls. T.BMD: total bone mineral density; T.BMC: total bone mineral capacity; DEXA: dual-energy X-ray absorptiometry; HOMA-IR: homeostasis model assessment of insulin resistance

Variable	Cases (n±50)			Controls (n±50)		
	Pearson's coefficient		P-value	Pearson's coefficient		P-value
T.BMC	-0.049	0.734		-0.091	0.528	
T.BMD	-0.070	0.629		-0.038	0.791	
T-score (bone)	0.296	0.037		0.131	0.366	
Z-score (bone)	0.244	0.081		0.177	0.218	

Table 8 shows the correlation of bone density with hs-CRP in cases and controls. No significant correlation of hs-CRP was observed with T.BMC, T.BMD, T-score, and Z-scores. 

**Table 8:** Correlation of bone density with hs-CRP in cases and controls. T.BMD: total bone mineral density; T.BMC: total bone mineral capacity; hs-CRP: high-sensitivity C-reactive protein

Variable	Cases (n±50)			Controls (n±50)		
	Pearson's coefficient		P-value	Pearson's coefficient		P-value
T.BMC	-0.096	0.506		-0.091	0.528	
T.BMD	-0.10	0.466		-0.38	0.791	
T-score (bone)	0.15	0.280		0.131	0.366	
Z-score (bone)	0.13	0.361		0.177	0.218	

## Discussion

NAFLD, a leading cause of chronic liver disease in the Western world, is expected to become the primary indication for liver transplantation by 2020. Mortality in NAFLD results from liver failure, metabolic complications, and cardiovascular issues. While Western studies consistently show higher mortality and morbidity in NAFLD patients, there is limited literature on NAFLD in India [11]. Population-based studies in India report NAFLD prevalence ranging from 8.7% to 32%, correlating with the metabolic syndrome affecting up to one-third of the population [12,13]. However, there's sparse data on the link between NAFLD, vitamin levels, and BMD in Asian Indians [14]. The rise of obesity, diabetes, and sedentary lifestyles in India contributes to an increased burden of metabolic syndrome, NAFLD, and associated BMD consequences [15].

NAFLD, closely tied to abdominal obesity, dyslipidemia, hypertension, and type 2 diabetes, may pose a higher risk of osteoporosis [16]. Current literature inadequately explores the association between NAFLD and BMD, with few global studies investigating NAFLD as an independent risk factor for low BMD, none from India [17].

The study's relevance lies in addressing the growing metabolic syndrome prevalence in India, impacting NAFLD and adiposity. Managing metabolic syndrome components can mitigate mortality and morbidity. Adiposity, a common cause of osteoporosis, is linked to cardiovascular, neurological, and metabolic issues [18]. While considered a metabolic syndrome component, evidence is lacking to confirm NAFLD as an independent osteoporosis risk. This study aims to determine if NAFLD, identified through abdominal ultrasound, correlates with BMD measured by whole-body DEXA scan.

Among NAFLD patients, nearly equal gender representation was observed, with 84% categorized as overweight or obese and 16% having a normal BMI. About 32% met the International Diabetes Federation (IDF) criteria for metabolic syndrome [19]. Lipid profile findings indicated elevated triglycerides (52%) and LDL levels above 100 mg/dL (54%) in NAFLD patients. Low HDL was present in 40%. NAFLD severity varied, with 60% having mild, 38% moderate, and one patient severe NAFLD among 50 patients.

Compared to non-NAFLD, NAFLD patients had significantly higher BMI, with obesity prevalence at 38% vs. 2%. While waist and hip circumferences were similar, waist-to-hip ratio was significantly higher in NAFLD (0.98±0.12 vs. 0.92±0.08, p=0.009). Skin fold thickness at various sites was higher in NAFLD, along with increased fat percentage and fat mass measured by bioimpedance analysis. However, FFM and TBW were comparable in both groups.

Fasting insulin levels were higher in NAFLD (8.9 vs. 5.7, p=0.08), and impaired fasting glucose prevalence was non-significantly higher (10% vs. 2%, p=0.204). Glucose tolerance test results and HOMA-IR were significantly higher in NAFLD. Subclinical inflammation, measured by hs-CRP and high ESR, was significantly more prevalent in NAFLD. These findings aligned with a study by Dayal et al. in India, showing elevated hs-CRP levels in overweight and obese children. Fasting insulin levels above 10 µIU/mL were more common in NAFLD (42% vs. 26%, p=0.09). The prevalence of impaired fasting glucose was higher in the NAFLD group, though non-significant (10% vs. 2%, p=0.204), possibly influenced by the small sample size and diabetes exclusion criterion [20].

In the study assessing BMD, NAFLD patients exhibited significantly lower BMD (1.09 vs. 1.15, p=0.02) and T-score (-0.244 vs. -0.346, p=0.0357) compared to non-NAFLD controls, with a higher prevalence of osteopenia in NAFLD (50% vs. 28%, p=0.039). These findings mirrored studies in obese adolescents, obese children, and adults reporting lower BMD in NAFLD groups [8,11,14].

In our study, whole-body DEXA scans revealed a significantly lower BMD in NAFLD cases than non-NAFLD controls (1.09 vs. 1.15, p=0.02). The T-score of NAFLD patients was also significantly lower (-0.244 vs. -0.346, p=0.0357), with a higher prevalence of osteopenia in NAFLD patients (50% vs. 28%, p=0.039). These findings align with studies by Pirgon et al. in obese adolescents, Pardee et al. in obese children, and Cui et al. in adult males and postmenopausal women, all reporting lower BMD in NAFLD groups [8,14,21].

A notable positive correlation was found between the T-score of bone and HOMA-IR in the NAFLD group (r=0.296, p=0.037). Further, a significant positive correlation was observed between fasting insulin levels and total fat percentage (r=0.281, p=0.005). No other noteworthy correlations were identified among the observed parameters.

Limited data exists on the correlation between NAFLD and BMD and vitamins A, C, and E in the Asian Indian population. Our study employed DEXA scans, considered the most appropriate method, to assess the percentage of body fat and BMD. Notably, there is only one other study in Asian Indians that evaluated BMD with NAFLD using DEXA scans at the hip and lumbar spine [9,22,23].

The study has several limitations that should be considered. Firstly, the sample size was relatively small due to time constraints. Additionally, NAFLD diagnosis relied on ultrasonography instead of liver biopsy, preventing the determination of the exact histological stage of the disease. Fasting insulin levels and HOMA-IR were utilized as surrogate markers for insulin resistance, deviating from the gold standard hyperinsulinemic-euglycemic clamp study. Moreover, the study did not account for vitamin intake through dietary methods, and data on physical activity were not included in the analysis, adding potential sources of variability to the findings. These limitations highlight areas for improvement and suggest caution in generalizing the results.

## Conclusions

This case-control study provides valuable insights into the association between NAFLD and BMD in the Asian Indian population. The findings indicate that NAFLD is significantly associated with lower BMD and a higher prevalence of osteopenia. The study also highlights correlations between insulin resistance, fat percentage, and bone health in NAFLD patients. However, the limitations of the study, including the small sample size and reliance on surrogate markers for insulin resistance, should be acknowledged. Further research with larger sample sizes, histological confirmation of NAFLD, and consideration of dietary and lifestyle factors is warranted to better understand the complex interplay between NAFLD, metabolic factors, and bone health in this population.
